# Diagnosis and Management of Heat-related Illness: Clinical and Molecular Perspectives

**DOI:** 10.14789/ejmj.JMJ25-0024-R

**Published:** 2025-11-18

**Authors:** JULIE HELMS, KENTA KONDO, KUNISHIKO NAGAKARI, TOSHIAKI IBA

**Affiliations:** 1 Strasbourg University, Medicine Faculty, University Hospital Federation (FHU) TARGET, Strasbourg University Hospital, Medical Intensive Care Unit NHC, Strasbourg, France; 1 Strasbourg University, Medicine Faculty, University Hospital Federation (FHU) TARGET, Strasbourg University Hospital, Medical Intensive Care Unit NHC, Strasbourg, France; 2 INSERM (French National Institute of Health and Medical Research) UMR 1260, Regenerative Nanomedicine (RNM), FMTS, Strasbourg, France; 2 INSERM (French National Institute of Health and Medical Research) UMR 1260, Regenerative Nanomedicine (RNM), FMTS, Strasbourg, France; 3 Department of Emergency and Disaster Medicine, Juntendo University Graduate School of Medicine, Tokyo, Japan; 3 Department of Emergency and Disaster Medicine, Juntendo University Graduate School of Medicine, Tokyo, Japan; 4 Department of Surgery, Juntendo University Urayasu Hospital, Chiba, Japan; 4 Department of Surgery, Juntendo University Urayasu Hospital, Chiba, Japan; 5 Faculty of Medical Science, Juntendo University, Chiba, Japan; 5 Faculty of Medical Science, Juntendo University, Chiba, Japan

**Keywords:** heat stroke, damage-associated molecular patterns, cell death, mitochondria, leukocyte, organ dysfunction

## Abstract

Heat-related illness (HRI) represents a growing public health challenge in the context of global warming. Ranging from mild symptoms such as heat cramps to life-threatening heatstroke, HRI requires prompt recognition and appropriate management to reduce morbidity and mortality. The diagnosis of HRI is primarily based on clinical symptoms but is supported by laboratory evaluation, especially in severe cases. Heatstroke, the most critical form, is defined by core body temperature ≥ 40°C and central nervous system dysfunction. In addition to conventional laboratory markers, recent advances in molecular biology have identified several biomarkers, such as heat shock proteins, HMGB1 (High Mobility Group Box 1), and mitochondrial DNA, which may improve early and accurate detection and prognostication. This review summarizes the diagnostic approach to HRI, highlighting key clinical findings, laboratory assessments, and emerging molecular markers.

## Introduction

Heat-related illness (HRI) refers to disorders that occur when the body fails to effectively dissipate excess heat during exposure to extreme environmental temperatures or intense physical exertion^1)^. With increasing global temperatures and more frequent heatwaves, the incidence of HRI is increasing significantly, especially among vulnerable populations such as the elderly, children, individuals with chronic diseases, and outdoor workers^2, 3)^. Early recognition and diagnosis are critical in reducing complications and improving survival, particularly in cases of heatstroke, which is associated with high mortality and multiorgan failure^4, 5)^. While the diagnosis of HRI has traditionally relied on clinical features, advancements in laboratory diagnostics and molecular research have provided deeper insights into its pathophysiology^6, 7)^. This review outlines a comprehensive diagnostic approach, integrating classical clinical criteria with emerging biomarkers and laboratory tests.

## Clinical classification and presentation

HRI is classified as a continuum of severity, ranging from mild to life-threatening conditions. It begins with heat cramps, characterized by painful muscle spasms during exertion. Heat exhaustion follows, marked by dehydration, weakness, dizziness, and profuse sweating, with preserved mental status. The most severe form is heatstroke, defined by a core body temperature ≥ 40°C and central nervous system dysfunction, such as confusion, seizures, or coma^1, 8)^. Heatstroke is further divided into classic (non-exertional) and exertional types^9)^. This classification aids in prompt recognition, risk stratification, and timely intervention to prevent progression to critical organ damage and death^10)^.

### Heat cramps

Heat cramps are painful, involuntary muscle spasms typically occurring in the legs, arms, or abdomen during or shortly after intense physical exertion in hot environments. These are often associated with electrolyte imbalances, particularly sodium loss due to sweating. Body temperature is usually normal or only mildly elevated. (Table 1)

**Table 1:** Classification of the heat-related illness

Clinical findings	Heat cramps	Heat syncope	Heat exhaustion	Heatstroke
CNS dysfunction	None	Brief syncope	None	Present (confusion, coma, seizures)
Core temp (°C)	Normal or mild elevation	Normal	Elevated, < 40°C	Elevated, ≥ 40°C
Other features	Muscle cramps	Transient LOC	Fatigue, nausea, dizziness	Multiorgan involvement
Skin	Moist	Cool, pale	Moist	Dry/hot (classic) or moist (exertional)

CNS: central nervous system, LOC: loss of consciousness

### Heat syncope

Heat syncope is a transient loss of consciousness caused by peripheral vasodilation, reduced venous return, and subsequent cerebral hypoperfusion. It usually occurs in individuals standing still in hot environments. Syncope is typically self-limited and resolves with rest and hydration.

### Heat exhaustion

Heat exhaustion presents with non-specific symptoms such as fatigue, headache, nausea, dizziness, hypotension, and tachycardia. Core temperature is elevated but generally remains below 40°C. The patient remains neurologically intact. It reflects the body's inability to sustain adequate thermoregulation and circulatory stability.

### Heatstroke

Heatstroke is the most severe form of HRI and is defined by a core temperature ≥ 40°C accompanied by central nervous system (CNS) dysfunction. Symptoms include confusion, agitation, seizures, or coma. It may progress to multiorgan failure involving the liver, kidneys, coagulation system, and cardiovascular function. Heatstroke is classified into two subtypes:

a. Classic (non-exertional) heatstroke: Occurs in elderly or chronically ill individuals during prolonged exposure to high ambient temperatures.

b. Exertional heatstroke: This affects healthy individuals, particularly athletes or military personnel, who are undergoing intense physical activity in hot environments.

## Diagnostic approach

The diagnostic approach to HRI begins with a thorough history of heat exposure, exertion level, and symptom onset, followed by assessment of vital signs and core body temperature, preferably via rectal measurement^11)^. Clinical features such as confusion, dry skin, and hypotension suggest progression to heatstroke^12)^. Laboratory evaluation includes serum electrolytes, renal and liver function tests, coagulation profile, creatine kinase, and arterial blood gases to assess organ dysfunction and complications like rhabdomyolysis or disseminated intravascular coagulation^5)^. Emerging biomarkers such as circulating mitochondrial DNA and heat shock proteins may provide early diagnostic and prognostic value in severe cases^13)^.

### History

An accurate clinical history is essential. Key factors include:

・Recent exposure to elevated ambient temperatures or strenuous physical activity

・Use of medications that impair thermoregulation (e.g., anticholinergics, diuretics, beta-blockers)

・Hydration status and access to cooling measures

・Underlying medical conditions that may predispose to HRI (e.g., diabetes, cardiovascular disease)

### Physical examination

Physical examination should include:

・Core body temperature: Rectal measurement is the most accurate

・Mental status: Neurological assessment is critical to distinguish heat exhaustion from heatstroke

・Skin findings: Dry, hot skin (classic heatstroke) vs. moist skin (exertional heatstroke)

・Vital signs: Hypotension, tachycardia, and tachypnea are frequently observed

### Laboratory investigations

Laboratory testing is essential for assessing severity, organ dysfunction, and guiding management.

・Hematology: Complete blood count (CBC) may show hemoconcentration, leukocytosis, or thrombocytopenia.

・Biochemistry and electrolytes: Sodium, potassium, chloride, calcium, and phosphate disturbances are standard tests.

・Renal function: Elevated BUN and creatinine indicate acute kidney injury (AKI) due to hypovolemia or rhabdomyolysis.

・Liver enzymes: aspartate aminotransferase (AST) and alanine aminotransferase (ALT) are frequently > 1000 IU/L in severe cases, reflecting hepatocellular injury. Elevated levels of bilirubin are more specific for liver dysfunction than transaminases alone.

・Muscle Injury: Creatine kinase (CK) level is often elevated > 10,000 IU/L in exertional heatstroke, indicating rhabdomyolysis.

・Myoglobinuria: Can be detected on urinalysis and may lead to acute tubular necrosis.

・Coagulation: prothrombin time (PT)/activated partial thromboplastin time (aPTT) and international normalized ratio (INR) are prolonged in cases of disseminated intravascular coagulation (DIC). In addition, D-dimer and fibrin degradation products (FDP) support the diagnosis of systemic coagulation activation.

・Blood gas analysis: Typically reveals metabolic acidosis and lactic acidemia. Elevated levels of serum lactate suggest tissue hypoperfusion.

・Cardiac markers: Troponin and brain natriuretic peptide (BNP) may be elevated, indicating myocardial stress or injury^14)^ (Table 2).

**Table 2:** Key laboratory findings in heatstroke

Parameter	Typical changes
CBC	Hemoconcentration, leukocytosis, thrombocytopenia
Electrolytes	Hyponatremia, hypo-/hyperkalemia
BUN/creatinine	Elevated (AKI from dehydration or rhabdomyolysis)
AST/ALT	Often >1000 IU/L (hepatic injury)
Total bilirubin	Elevated (reflecting liver dysfunction)
CK	Elevated, often >10,000 IU/L (rhabdomyolysis)
PT/aPTT, D-dimer	Prolonged PT/aPTT, elevated D-dimer (DIC)
Arterial blood gas	Metabolic acidosis with elevated lactate
Urinalysis	Myoglobinuria, hematuria
Cardiac markers (Troponin, BNP)	Elevated in some severe cases

CBC: cell blood count, BUN: blood urea nitrogen, AST: aspartate aminotransferase, ALT: alanine aminotransferase, CK: creatine kinase, PT: prothrombin time, aPTT: activated partial thromboplastin time, BNP: brain natriuretic peptide, AKI: acute kidney injury, DIC: disseminated intravascular coagulation

## Organ-specific involvement

Heatstroke can lead to widespread organ dysfunction due to hyperthermia-induced cellular injury, systemic inflammation, and microvascular disturbances. The CNS is particularly vulnerable, with manifestations including delirium, seizures, or coma, reflecting neuronal injury and cerebral edema^15)^. The cardiovascular system may show hypotension, arrhythmias, and myocardial injury due to volume depletion and thermal stress^16)^. Renal failure is common and often results from rhabdomyolysis, dehydration, and direct tubular injury^17, 18)^. Liver dysfunction, including acute hepatic failure, may occur due to ischemia, inflammation, and mitochondrial damage^19)^. The coagulation system is frequently impaired, leading to DIC and hemorrhagic complications^20)^. In the gastrointestinal tract, splanchnic hypoperfusion and endothelial injury can cause ischemia, mucosal barrier disruption, and bacterial translocation^21)^. The immune system is activated initially, but prolonged hyperthermia can lead to leukocyte exhaustion and immunosuppression, increasing the risk of secondary infections^22)^.

### Central nervous system

Central nervous system (CNS) dysfunction is the hallmark of heatstroke. Neurological symptoms may be subtle early on and progress to coma or seizures. Electroencephalogram and neuroimaging (CT or MRI) may be necessary to rule out structural or infectious causes.

### Renal

Heatstroke-associated rhabdomyolysis contributes to myoglobin-induced AKI. Serial creatinine monitoring and urine output measurement are essential.

### Hepatic

Severe hepatic injury may occur, with elevations in AST (aspartate aminotransferase) and ALT (alanine aminotransferase) and rising bilirubin.

### Coagulation

DIC is a common complication. Serial monitoring of platelets, prothrombin time (PT)/activated partial thromboplastin time (aPTT), fibrinogen, and D-dimer is recommended.

## Differential diagnosis

The differential diagnosis of heat-related illness, particularly heatstroke, includes conditions that present with hyperthermia and altered mental status. Sepsis, neuroleptic malignant syndrome, serotonin syndrome, and malignant hyperthermia must be considered, as all can cause high fever and multiorgan dysfunction^23, 24)^. Thyrotoxic crisis and anticholinergic toxicity also mimic heatstroke, with hyperthermia and autonomic instability^25)^. Key distinguishing features include medication history, infectious sources, muscle rigidity, and laboratory markers such as creatine kinase levels and leukocyte profiles^26, 27)^. Accurate diagnosis relies on context, including environmental exposure and exertion, and is critical for appropriate management and preventing further deterioration.

The following conditions may mimic heatstroke and should be ruled out:

・Sepsis: Often indistinguishable initially; fever, leukocytosis, and hypotension may overlap.

・Neuroleptic malignant syndrome (NMS): Associated with antipsychotic use.

・Serotonin syndrome: Hyperthermia, clonus, agitation.

・Thyroid storm: Requires thyroid function testing.

・Malignant hyperthermia: Usually anesthetic-induced, rapid onset.

・Drug intoxications: Anticholinergic, sympathomimetic.

## Molecular and emerging biomarkers

Molecular and emerging biomarkers offer valuable insights into the pathogenesis and prognosis of heatstroke. HSPs, particularly HSP70, are upregulated in response to thermal stress and may reflect cellular injury or protection^28)^. Circulating mtDNA and cell-free nuclear DNA act as damage-associated molecular patterns (DAMPs), triggering systemic inflammation^29)^. Elevated levels of HMGB1 and interleukin-6 (IL-6) correlate with disease severity^30)^. Additionally, mtRNAs have been implicated in immune modulation during heatstroke^31)^. These biomarkers may aid early diagnosis, risk stratification, and targeted therapy development.

### Heat shock proteins (HSPs)

HSP70 and HSP90 are stress-induced chaperones that increase during hyperthermia.

Their serum levels reflect tissue stress and injury severityHSP70 may also have protective roles in cellular repair^32)^.

### Proinflammatory cytokines

IL-1β, IL-6, and tumor necrosis factor (TNF)-α levels rise sharply in heatstroke, reflecting systemic inflammatory response syndrome (SIRS)^33)^. These cytokines contribute to endothelial dysfunction and coagulopathy.

### High-mobility group box 1 (HMGB1)

HMGB1 is a nuclear protein released from necrotic cells. It acts as a DAMP that triggers toll-like receptor (TLR) signaling and amplifies inflammation. Elevated levels are associated with poor prognosis^34)^.

### Mitochondrial DNA (mtDNA)

Cell-free mtDNA is a potent DAMP that activates immune receptors. It is associated with systemic inflammation, coagulation activation, and endothelial injury^35)^.

### Endothelial injury markers

Soluble thrombomodulin, syndecan-1, and decreased protein C activity are markers of endothelial damage^36)^. These markers help stratify the risk of coagulopathy and organ failure.

### Neutrophil extracellular traps (NETs)

NETs comprise DNA, histones, and granular enzymes released by activated neutrophils. NET formation contributes to microvascular thrombosis and DIC. Measurement of cell-free DNA and myeloperoxidase (MPO)-DNA complexes may serve as biomarkers^37)^.

## Prognostic scoring and clinical guidelines

While there is no heatstroke-specific scoring system, tools from critical care practice are commonly used, such as SOFA (Sequential Organ Failure Assessment) and APACHE (acute physiology and chronic health evaluation) II, which are often applied to predict outcomes^5)^. Elevated scores correlate with increased risk of mortality and multi-organ failure. Some studies propose heatstroke-specific indices incorporating core temperature, CNS dysfunction, and laboratory abnormalities (e.g., liver enzymes, creatine kinase)^38, 39)^. Clinical guidelines from bodies such as the Japanese Association for Acute Medicine (JAAM) and Exertional Heat Illness Consensus Statements emphasize early cooling, organ support, and tailored resuscitation^40)^.

・SOFA score: Assesses multi-organ dysfunction and correlates with mortality.

・JAAM DIC score: Useful for tracking coagulopathy.

・Bouchama's criteria^1)^: Define heatstroke as core temperature ≥ 40°C with CNS abnormalities and no other cause.

## Recent advances in the research of pathophysiology

Heat-related illness, particularly heatstroke, involves complex molecular mechanisms beyond thermoregulatory failure. Hyperthermia induces cellular damage and inflammation, through the release of DAMPs such as mtDNA and HMGB1, which activate immune responses via pattern recognition receptors like TLRs, amplifying systemic inflammation^29-31)^. HSPs, especially HSP70, are upregulated in response to thermal injury, serving dual roles as protective chaperones and proinflammatory mediators^28, 32)^. Additionally, proinflammatory cytokines (e.g., IL-6, TNF-α) and NETs contribute to endothelial injury, coagulation activation, and multiorgan dysfunction^33, 37)^. These molecular alterations underlie the systemic inflammatory response and coagulopathy observed in severe HRI, and emerging biomarkers like circulating mtDNA, HSPs, and NET components are under investigation for diagnosis and prognosis^29, 35, 36)^.

## Treatment of heat-related illness

The cornerstone of treatment for heat-related illness, particularly heatstroke, is rapid cooling and organ support. The primary goal is to reduce core body temperature to below 38.9°C (102°F) as quickly as possible to prevent further cellular and organ damage. The most effective method is cold water immersion, especially in exertional heatstroke, where cooling rates of 0.15-0.20°C per minute can be achieved^10, 41, 42)^. If immersion is not feasible, alternative methods such as evaporative cooling (spraying the body with water while applying fan air), ice packs to axillae and groin, or cooling blankets may be used^1, 43)^. In cases of severe exertional heatstroke unresponsive to conventional cooling methods and associated with cardiovascular or respiratory failure, extracorporeal membrane oxygenation (ECMO) can be used not only for organ support but also as a rapid cooling modality. Case reports suggest that extracorporeal cooling through ECMO circuits can lower core temperature efficiently while maintaining hemodynamic stability, potentially improving neurologic outcomes in select patients.

Fluid resuscitation with isotonic crystalloids is essential to correct dehydration, support circulation, and maintain urine output. Electrolyte imbalances should be monitored and corrected. In severe cases, multiorgan dysfunction may require advanced supportive care, including mechanical ventilation for respiratory failure, renal replacement therapy for acute kidney injury, and vasopressors if hypotension persists despite adequate volume resuscitation^4, 44)^.

Coagulation abnormalities such as DIC may require transfusions (fresh frozen plasma, platelets) and potentially antithrombin supplementation^45)^. Close monitoring of coagulation parameters is recommended.

Emerging therapies target inflammation and mitochondrial dysfunction, including agents that modulate cytokine production or stabilize mitochondrial membranes, though these are still under investigation^46)^.

Prompt recognition and initiation of aggressive cooling remain the most critical determinants of survival. Delays in cooling are strongly associated with increased morbidity and mortality.

## Conclusion

Heat-related illness, especially heatstroke, represents a growing threat in the era of climate change. Prompt diagnosis based on clinical features remains the cornerstone, but laboratory tests are critical for assessing organ involvement. Emerging molecular biomarkers such as HSPs, HMGB1, mtDNA, and NETs offer promise for improved diagnosis, prognostication, and therapeutic targeting. Integrating traditional clinical assessment with molecular diagnostics may enhance outcomes in patients with severe HRI.

## Author contributions

JH and TI wrote the manuscript. KK and KN revised the text. All authors read and approved the final manuscript.

## Conflicts of interest statement

J. Helms and T. Iba, members of the JMJ Editorial Board, were not involved in the peer review or decision-making process for this paper. The authors declare that they have no conflict of interest.
